# State Society

**Published:** 1871-11

**Authors:** 


### Editorial.

### STATE SOCIETY.

On the 5th of December, prox., will be held the annual meeting of the Ohio State Dental Society, at Columbus, and we trust there will be a large and efficient meeting, one that shall clearly make manifest that the spirit of progress, so fully shown in former meetings is onward and upward. Let no one who has hitherto put his hand to the work in this direction turn back—but take courage and gain strength, for accomplishing more than ever before. The profession in Ohio has a great work before it, and so soon as the present instrumentalities for good are in proper exercise, and accomplishing their work with facility, new instrumentalities and new methods will present themselves. When the present fields are under proper cultivation, new ones will be opened up.

The progressive spirit of the age, pervades the dental profession, not less than other departments of human occupation, and upon those who are actors, new duties are continually devolving.

Let every member come up to this society fully impressed with the responsibility that rests upon him, and with a fixed resolution to fulfill his mission. Let the principles and influences that pertain to the profession, be so molded and directed, as to secure the greatest good to all concerned.

We hope to greet every good member of the profession in the State at this meeting, and let it be a time of encouragement, of adding strength and power, not only to individuals, but to the profession of our State. T.

